# Adaptation and validation of the instrument Clinical Learning Environment and Supervision for medical students in primary health care

**DOI:** 10.1186/s12909-016-0809-8

**Published:** 2016-12-01

**Authors:** Eva Öhman, Hassan Alinaghizadeh, Päivi Kaila, Håkan Hult, Gunnar H. Nilsson, Helena Salminen

**Affiliations:** 1 Division of Family Medicine, Department of Neurobiology Care Sciences and Society, Karolinska Institutet, Alfred Nobels allé 23, Huddinge, SE 141 83 Sweden; 2 Linköping University, Linköping, Sweden; 3 Academic primary health care centre (APC), County Council of Stockholm, Alfred Nobels allé 10, Huddinge, Sweden

**Keywords:** Medical students, Primary health care, Clinical learning environment, Validation

## Abstract

**Background:**

Clinical learning takes place in complex socio-cultural environments that are workplaces for the staff and learning places for the students. In the clinical context, the students learn by active participation and in interaction with the rest of the community at the workplace. Clinical learning occurs outside the university, therefore is it important for both the university and the student that the student is given opportunities to evaluate the clinical placements with an instrument that allows evaluation from many perspectives. The instrument Clinical Learning Environment and Supervision (CLES) was originally developed for evaluation of nursing students’ clinical learning environment.

The aim of this study was to adapt and validate the CLES instrument to measure medical students’ perceptions of their learning environment in primary health care.

**Methods:**

In the adaptation process the face validity was tested by an expert panel of primary care physicians, who were also active clinical supervisors. The adapted CLES instrument with 25 items and six background questions was sent electronically to 1,256 medical students from one university. Answers from 394 students were eligible for inclusion. Exploratory factor analysis based on principal component methods followed by oblique rotation was used to confirm the adequate number of factors in the data.

Construct validity was assessed by factor analysis. Confirmatory factor analysis was used to confirm the dimensions of CLES instrument.

**Results:**

The construct validity showed a clearly indicated four-factor model.

The cumulative variance explanation was 0.65, and the overall Cronbach’s alpha was 0.95. All items loaded similarly with the dimensions in the non-adapted CLES except for one item that loaded to another dimension. The CLES instrument in its adapted form had high construct validity and high reliability and internal consistency.

**Conclusion:**

CLES, in its adapted form, appears to be a valid instrument to evaluate medical students’ perceptions of their clinical learning environment in primary health care.

**Electronic supplementary material:**

The online version of this article (doi:10.1186/s12909-016-0809-8) contains supplementary material, which is available to authorized users.

## Background

Clinical learning occurs in a multidimensional environment, a place where the patient gets help, a workplace for the staff and a learning place for the student, a place containing physical, social and educational dimensions [1, 2]. Learning in real workplaces through encounters with patients and their families, other health care professions and students is an essential part of medical education. The main purpose of learning in real workplaces is to give the student opportunities to translate theoretical knowledge into practice, to provide early professional contact, and to allow the student to build an identity as a professional [3].

Students who have their clinical placement at a primary health care (PHC) centre meet patients of several ages with various complaints, a context which is usually perceived as stimulating by the students [2, 4]. At the PHC centre, the student is also given the opportunity to see the patient as an individual [4]. The student has the possibility to interact with the supervisor, other students and professions [2, 5]. This is important according the theory of social learning, where the engagement and participation with others in the community is the basis of learning, “learning as social participation” [6]. The role of the supervisor is identified as a key factor for a meaningful learning experience [2]. Students describe how a good relationship between a student and a supervisor is characterised by a positive attitude, openness, trust, and an atmosphere that allows the student to ask questions and thus reveal gaps of knowledge [3, 4, 7, 8]. Supervision at a PHC centre is often organized as a one-to-one relationship where a student has an appointed doctor as supervisor. It has been reported that one-to-one relationships allow the supervisor to observe and support the student’s learning process more individually and gradually let the student take care of patients more independently [2]. The clinical practice in PHC occurs outside the university and the student is dependent not only on the supervisor but also on the rest of the staff, individuals from different professions [2, 5]. This multifaceted learning environment requires a multifaceted instrument for its evaluation.

A systematic review suggested in 2010 that the Dundee Ready Education Environment Measure (DREEM), the Postgraduate Hospital Educational Environment Measure (PHEEM) and the Clinical Learning Environment and Supervision (CLES) are the most suitable instruments for undergraduate medical, postgraduate medical and nursing education [9]. DREEM covers several aspects of an educational environment at university as a learning environment [10, 11]. PHEEM is an internationally used instrument for measuring the educational climate of postgraduate hospital environments [12]. The PHEEM instrument has three subscales: perceptions of role autonomy, perceptions of teaching and perceptions of social support [12]. CLES is an instrument originally developed for the evaluation of the clinical learning environment of nursing students [13].

CLES + T, the instrument developed from the original CLES instrument, is currently used since 2007 for evaluation of the quality of the clinical learning environment in Hospital District of Helsinki and Uusimaa, in Finland [14].

After the BEME review was published two new instruments for measuring clinical teaching environment for medical students has been created; Manchester Clinical Placement Index (MCPI) 2012 [15] and the Undergraduate Clinical Education Environment Measure (UCEEM) 2013 [16] Both instruments were validated for evaluation of the clinical learning environment of medical students in hospitals and in PHC. An instrument that could be used for evaluation of the clinical learning environment of both medical students and other students from other health care professions in PHC would give new opportunities to create systems for quality control and give more nuanced feedback to both the university and the clinical sites. Items in CLES are relatively independent of profession. Considering this and the results from previous validation studies, the CLES instrument was chosen for this study.

The aim of this study was to adapt and validate the CLES instrument to measure medical students’ perceptions of their learning environment in PHC.

## Methods

### Context and participants

Medical students at Karolinska Institutet, Stockholm Sweden, had clinical placements in PHC for 10 weeks, spread over nine semesters, during their clinical education. The period at the PHC centre varied from two to seven days per semester. The students had their clinical placements at 152 different PHC centres, depending on which semester the students were in their education.

The adapted CLES instrument was sent as a web-based questionnaire to 1,256 medical students in spring 2012. The web programme showed who had responded to the survey but not what they had answered. A reminder was sent one week after the first inquiry to the students who had not responded.

All items in the web questionnaire were mandatory. The entire CLES websurvey is available in Additional file 1.

### The CLES instrument

The original CLES instrument was created in 2002 as an instrument designed for evaluation of the clinical learning environment of nursing students in Finland [13]. CLES was developed from extensive literature reviews, audits and the previous work of the Finnish research group. The CLES instrument contained 27 items and was divided into five sub-dimensions. Its face validity was tested by an expert panel. CLES was then tested by Finnish nurse students (*n* = 162) and the instrument was assessed by nine clinical teachers. Exploratory factor analysis was used to analyses the construct validity and the reliability was ranged from 0.94 to 0.73 tested by Cronbach’s Alpha [13].

CLES was further developed to CLES + T in 2008, containing a new dimension: ‘Role of nurse teacher’. The other dimensions were ‘Supervisor relationship’, ‘Pedagogical atmosphere at the ward’, ‘Leadership style of the ward manager’ and ‘Premises of nursing on the ward’ [17]. The CLES instrument has 25 items and utilizes a 5-point Likert scale: (1) Fully disagree, (2) Disagree to some extent, (3) Neither agree nor disagree, (4) Agree to some extent, and (5) Fully agree. The CLES + T instrument has been tested by several psychometric tests [18–20] CLES + T has been validated for evaluation of the clinical learning environment for nursing students in PHC in Sweden [20].

### Adaptation and assessing the face and content validity of the instrument

The original CLES instrument was developed to measure nursing students’ perceptions of their learning environment in hospital units. The original CLES instrument was developed with the added dimension ‘Role of nursing teacher’ and became the CLES + T instrument.

The authors of this article decided to remove the dimension ‘Role of nursing teacher’ from the original instrument for the purpose of this study because there is no comparable role of a teacher at a PHC centre to facilitate the medical students’ learning. Some items and terms such as ‘ward’ and ‘ward manager’ (WM) in the original CLES were not applicable to PHC and have been replaced with ‘PHC centre’ and ‘manager at the PHC centre (Table 1). The dimension in the original instrument ‘Premises of nursing on the ward’ was not suitable since nursing philosophy not is applicable in the context of medical students in PHC and therefore it was changed by the authors of the article to ‘Premises for the patient’. In order to minimize the risk of response biases, the four items in the dimensions “Premises for the patient” were adapted by using meanings that were appropriate for respondents in our sample. Item 31) “The ward´s nursing philosophy was clearly defined” was adapted to “The PHC Centre has a clearly defined vision and a mission statement for patient care that is clearly described”. Item 32) “Patients received individual nursing care” was adapted to “patients received individualised care”. Item 33) “There were no problems in the information flow related to patients care” was adapted to “There were no problems in the information flow related to patient care (Discussion about individual patients and the transmission of information about individual patient cases to other colleagues and team members were handled respectfully) “. Item 34) “Documentation of nursing (e.g. nursing plans, daily recording of nursing procedures etc.) was clear” was adapted to “Documentation of patient care (e.g. medical records and other medical procedures etc.) was clear.” Those and others adaptation are also described in Table 1.

**Table 1:** Adaptation of the CLES items to the context of medical students in PHC

The CLES items version 2008	The CLES items adapted for medical students
Overall I am satisfied with the supervision I received	Overall, I am satisfied with the supervision I received at the PHC centre
I felt comfortable going to the ward at the start of my shift	I felt comfortable going to the PHC centre every day of my practice
During staff meetings (e.g., before shifts) I felt comfortable taking part in the discussion	During staff meetings I felt comfortable taking part in the discussions
There was a positive atmosphere on the ward	There was a positive atmosphere at the PHC centre
There were sufficient meaningful learning situations on the ward	There were sufficient meaningful learning situations at the PHC centre
The ward can be regarded as a good learning environment	The PHC centre can be regarded as a good learning environment
The WM regarded the staff on her/his ward as a key resource	The manager of the PHC centre regarded the staff at their PHC centre as a key resource
The WM was a team member	The manager of the PHC centre was a team member
Feedback from the WM could easily be considered a learning situation	Feedback from the manager of the PHC centre could easily be considered as a learning situation.
The ward’s nursing philosophy was clearly defined	The PHC centre has a clearly defined vision and a mission statement for patient care that is clearly described
Patients received individual nursing care	Patients received individualised care
There were no problems in the information flow related to patients care	There were no problems in the information flow related to patient care (Discussions about individual patients and the transmission of information about individual patient cases to other colleagues and team members were handled respectfully)
Documentation of nursing (e.g., nursing plans, daily recording of nursing procedures etc.) was clear	Documentation of patient care (e.g., medical records and other medical procedures etc.) was clear

After the adaptation process, an expert panel assessed the face- and content validity. The expert panel consisted of five clinically active primary care physicians, and clinical teachers in PHC. All adaptation was performed in collaboration with Mikko Saarikoski, the creator of the original CLES instrument.

### Definitions

The terms supervisor, tutor, and mentor have been used slightly differently in different studies. In this study, the term ‘supervisor’ was used to describe a person with a formal mandate to supervise students. The term ‘manager at the PHC centre’ was used to describe the person who is the manager of a single PHC centre.

### Statistics

The first step was to find out how well the adapted CLES measured the clinical learning environment for medical students in PHC, the *construct validity*. To assess the construct validity, with the aim to analyse the underlying structure of all items and control for grouping tendency, exploratory factor analysis was used. The suitability of the data was confirmed by the Kaiser-Meyer-Olkin (KMO) index of sampling adequacy and a significant Bartlett test of sphericity.

The *reliability and the internal consistency* of the instrument were controlled by using Cronbach´s alpha coefficient. Analysis of Cronbach´s alpha was performed to control for internal consistency.

Exploratory factor analysis based on principal component methods, followed by oblique rotation was used to confirm the number of factors. Oblique rotation was used with purpose to show associations among factors. The next step was to confirm if there was an adequate number of factors. Confirmatory factor analysis (CFA) based on the polychoric correlation was used to confirm if the four factors model was correct in the final step and chosen critical Fit value for acceptance were Root Mean Square Residual (RMSR), Standardized RMSR, Goodness of Fit Index (GFI), Adjusted Goodness of Fit Index (AGFI), Parsimony Goodness of Fit Index (PGFI) and Bentler-Bonett NFI.

The term factor is a statistical term and therefore factor was used in the statistical part of the methods section. In the discussion and in the tables, the word dimension was used.

All statistical analyses were performed using the SAS 9.3 software (SAS Institute, Cary, NC, USA).

## Results

A total of 394 students answered the questionnaire (mean age of 26 years, range 19–53 years); 63 % of the students were women and 37 % were men. Between 101 and 160 questionnaires were sent to nine different semesters and between 30 and 53 answers where received from each semester. All items in the web questionnaire were mandatory, so the questionnaires were answered fully and completely, and consequently the dataset contained no missing data.

### Construct validity

As shown in Table 2 of the 25 items in the adapted CLES loaded to the four factors with a loading above 0.5.

Almost all items, 23 out of 25, had loadings above 0.5. The lower limit was drawn at 0.3 for items loadings to factors. All items loaded similarly to those of the CLES from 2008, without the removed dimension ‘T’ [17] except for one item. In the original CLES, the item ‘*The ward’s nursing philosophy was clearly defined*’ loaded in the fourth dimension, ‘Premises of nursing on the ward.’ For the purpose of our study, this item was adapted and rephrased as ‘*The* PHC centre *has a clearly defined vision and a mission statement for patient care that is clearly described’* (see Table 1). This item moved to dimension 3, ‘Leadership style of the manager of the PHC centre.’

**Table 2:** CLES validation for medical students in PHC with exploratory factor analysis confirmed with confirmatory factor analysis. The table shows factor loadings for both EFA and CFA and results of Cronbach’s Alpha

Items nr^a^	Item^b^	EFA^c^	CFA^d^	Cronbach’s alpha^e^
D1	Supervisor relationship (Dimension)			
Item 1	My supervisor showed a positive attitude towards supervision	0.60	0.93	0.95
Item 2	I felt that I received individual supervision	0.50	0.85	0.95
Item 3	I continuously received feedback from my supervisor	0.61	0.84	0.95
Item 4	Overall I am satisfied with the supervision I received at the PHC centre	0.55	0.96	0.95
Item 5	The supervision was based on a relationship of equality and promoted my learning	0.83	0.89	0.95
Item 6	There was a mutual interaction in the supervisory relationship	0.86	0.92	0.95
Item 7	Mutual respect and approval prevailed in the supervisory relationship	0.87	0.90	0.95
Item 8	The supervisory relationship was characterized by a sense of trust	0.85	0.85	0.95
D2	Pedagogical atmosphere on the PHC centre			
Item 9	The staffs was easy to approach	0.53	0.84	0.95
Item 10	I felt comfortable going to the PHC centre every day of my practice	0.69	0.81	0.95
Item 11	During staff meetings I felt comfortable taking part in the discussions	0.44	0.62	0.95
Item 12	There was a positive atmosphere at the PHC centre	0.61	0.79	0.95
Item 13	The staff was generally interested in student supervision	0.57	0.84	0.95
Item 14	The staff learned to know the students by their personal names	0.77	0.65	0.95
Item 15	There were sufficient meaningful learning situations at the PHC centre	0.74	0.84	0.95
Item 16	The learning situation were multidimensional in terms of content	0.61	0.75	0.95
Item 17	The PHC centre can be regarded as a good learning environment	0.66	0.88	0.95
D3	Leadership style of the manager of the PHC centre			
Item 27	The manager of the PHC centre regarded the staff at their PHC centre as a key resource	0.81	0.82	0.95
Item 28	The manager of the PHC centre was a team member	0.87	0.68	0.95
Item 29	Feedback from the manager of the PHC centre could easily be considered as a learning situation	0.76	0.75	0.95
Item 30	The effort of individual employees was appreciated	0.65	0.74	0.95
Item 31	The PHC centre has a clearly defined vision and mission statement for the patient care that is clearly described	0.60	0.71	0.95
D4	Premises of the patient			
Item 32	Patients received individualised care	0.43	0.86	0.95
Item 33	There were no problems in the information flow related to patient care (Discussions about individual patients and the transmission of information about individual patient cases to other colleagues and team members were handled respectfully)	0.74	0.67	0.95
Item 34	Documentation of patient care (e.g. medical records and other medical procedures etc.) was clear	0.72	0.57	0.95

^a^Item numbered in the original CLES

^b^Item translated for PHC

^c^Exploratory Factor Analysis

^d^Second-order Confirmatory Factor Analysis

^e^Cronbach’s alpha when item was deleted

### Reliability and internal consistency

The rated adequacy of the sampling was 0.95 according to Kaiser-Meyer-Olkin analysis, which showed that it was appropriate to perform factor analysis on the data. The items clearly loaded to a four-factor model with a 65 % cumulative variance explanation. Eigenvalue for factor 1 was 11.88 showing a high grade of explanation of the variance in the factor. Eigenvalue for factor 2 was 2.14, 1.26 for factor 3 and for factor 4, 0.98. Proportion had highest value 0.48for factor 1, 0.09 for factor 2, 0.05 for factor 3, and 0.04 for 4.

The reliability of the instrument was estimated using Cronbach’s alpha, which measured how consistent items were within each factor. The internal consistency for the 25 items was found to be high, with an overall Cronbach’s alpha value of 0.95. Cronbach’s alpha was 0.91 for factor 1, 0.92 for factor 2, and 0.95 for factors 3 and 4. (Table 2).

These four dimensions were confirmed by CFA and RMSR = 0.06, SRMSR = 0.06, GFI = 0.99, AGFI = 0.99, PGFI = 0.89 and NFI = 0.99 all indicating a good fit.

For additional results see the Additional files containing:

Additional file 2: Table S3. The means and standard deviations of the items in the CLES instrument.

Additional file 3: Table S4. The frequency of the Likert scale answers of the items in the CLES instrument.

Additional file 4: Table S5. The inter-factor correlations of the CLES instrument.

## Discussion

The main finding of our study was that CLES, in its adapted form, appears to be a valid instrument for measuring medical students’ perceptions of the learning environment in PHC. The items clearly loading to a four-factor model indicated that CLES can be regarded as valid for this new target group and new context.

In our study one item, the item that highlights the perspective on the patient care moved from its original dimension ‘Premises for the patient’ to a new dimension. The item ‘*The PHC centre has a clearly defined vision and mission statement for patient care that is clearly described*’ loaded to the dimension ‘Leadership style of the clinical manager of the PHC centre’. This item might have changed its meaning in the adaptation process. It might be logical that the item moved to another dimension when the context was a PHC centre and the target group medical students. The medical students’ placement at the PHC centre varied between two to seven days per semester. The items in the dimension ‘Leadership style of the manager of the PHC centre’ might be difficult for medical students to answer adequately if they only had had a PHC placement for a few days. Longer clinical placements might enable more interaction with managers and other staff in the PHC.

Although both the context and the target audience are new, we found basically the same loading pattern as in one previous study [13] except for one item, which indicates the stability of the instrument in our learning environment.

The reason to choose CLES for adaptation and validation instead of using existing evaluation instrument, as for example DREEM or PHEEM, was the ambition to find a validated instrument suitable for medical students in the special clinical learning environment in PHC where students from other professions also have clinical placements. To be able to evaluate the learning environment of students from different professions, with the same instrument would facilitate the qualitative work and make it possible to compare the results. DREEM is one of the often used and recommended instruments but does not cover the aspects of the clinical learning environment [9–11]. PHEEM is a validated instrument developed for postgraduate hospital based junior doctors that is frequently used and that is translated to several languages, [12]. During the literature search for this study, we could not find studies for any of the instruments above that discussed the validation of the instrument DREEM or PHEEM to PHC. Therefore the choice to use CLES was made. One previous study has shown CLES to be valid for evaluation of the learning environment of nurse students in PHC [20]. There exist today two other instruments that have been created for evaluation of the medical students’ clinical learning environment in PHC. In 2012, the Manchester Clinical Placement Index (MCPI) was introduced [19]. The MCPI contains eight items with the opportunity for the student to comment freely on each of them, and the same items are used for both hospitals and PHC [15]. Compared to MCPI, CLES contains a larger number of items per dimension, which may increase the students’ possibility to describe their perception of the clinical learning environment in a more differentiated way. In 2013, the Undergraduate Clinical Education Environment Measure (UCEEM) was introduced, an instrument that contains 25 items that highlight many aspects of the clinical learning environment [16].

Neither MCPI nor UCEEM include the perspective of patient care. Patients are an indispensable part of medical students’ learning, but studies investigating the clinical learning environment of medical students rarely highlight the dimensions of the patient or the health care philosophy that prevails in the clinic. The CLES’s dimension ‘Premises for the patient’ may give the student a possibility to evaluate their perception of patient philosophy and the approach to the patient at the PHC centre.

As previous studies have shown – students learn by doing [3, 5] and the learning occurs in, and is influenced by, a social context [2, 5, 6, 21]. It has been shown in a study of Hendelman and colleagues that 72 % of the medical students have, sometime during their clerkship, witnessed a lapse in professionalism, carried out by for example physicians, nurse or other staff [22, 23].

As medical education in PHC takes place in a complex environment, occurring outside the university, it is necessary to make sure that it is possible for the student to evaluate and give expression from many different perspectives. Items in the dimension ‘Supervisor relationship’ and’Pedagogical atmosphere at the PHC centre’ in CLES cover both aspects of the relation between the student and supervisor and the pedagogical atmosphere at the PHC centre.

### CLES in the future

The items in CLES are relatively independent of profession, which could make the instrument suitable to be adapted for evaluation of several professions’ learning environment. An evaluation instrument that can be used to evaluate the clinical learning environment for students from multiple healthcare professions could be used to obtain a more complete picture of the clinical learning environment. It would also facilitate the comparison of units and clinics. The original CLES instrument for nursing educations has been adapted to local contexts [19, 24]. It is possible that similar adaptation needs to be made if the instrument is going to be introduced for medical students and students from other professionals in a wider context. In Finland, CLES + T is used by hospital and medical universities for quality control of the clinical learning environment [14]. As the Finnish example, there may be a large potential for the CLES instrument to be used for the evaluation of the clinical learning environment for several student professions at a PHC centre. The results of the evaluation could be used to compare different PHC centres` learning environment. Feedback can be given to both the supervisor, the PHC centre, the student and the medical university, thus ideally improving the quality of the clinical learning environment.

A quantitative measurement with CLES can give valid information about students’ perceptions, but also qualitative methods such as observations and interviews are needed for a deeper understanding of the clinical learning in PHC. A qualitative part was introduced to the version of CLES that was implemented for evaluation of the learning environment of medical students in PHC.

### Strengths and limitations

This study has both strengths and limitation. Students who answered the CLES questionnaire were all medical students who had recently had a clinical placement in PHC which could be considered as a strength.

The fact that all items in the adapted CLES were mandatory could be considered as both a strength and a limitation. The number of responses can be regarded as sufficient because all items were mandatory to answer. Each item was answered by 394 participants, and there were no missing values. The limitation of using mandatory items could be that if a student did not want to answer one of the items, they could not leave it and continue. This could possible make the student reluctant to continue and exit from the survey.

The CLES instrument was created in the early 2000s. Education and educational premises for clinical learning develop continuously over time, and new pedagogical methods might require other questions or items in order to adequately evaluate the clinical environment in future.

## Conclusion

Our study shows that the adapted CLES instrument, when used among medical students in PHC, had high construct validity with items clearly loading to a four-factor model. Our results showed both high reliability and high internal consistency. The CLES can be considered to be a promising tool for the evaluation of today’s learning environments for medical students in PHC.

## Additional files

Additional file 1: The Clinical Learning Environment and Supervision (CLES) in its adapted form, sent to medical students who had clinical practice in primary health care.
Additional file 2: Table S3. The means and standard deviations of the items in the CLES instrument.
Additional file 3: Table S4. The frequency of the Likert scale answers of the items in the CLES instrument.
Additional file 4: Table S5. The inter-factor correlations of the CLES instrument.

## Abbreviations

CLES: Clinical Learning Environment and Supervision
DREEM: Dundee Ready Education Environment Measure
MCPI: Manchester Clinical Placement Index
PHC: Primary Health Care
PHEEM: Postgraduate Hospital Educational Environment Measure
UCEEM: Undergraduate Clinical Education Environment Measure
