# Supplementary material for: Adaptation and validation of the instrument Clinical Learning Environment and Supervision for medical students in primary health care
Source: BMC Med Educ. 2016 Dec 1;16:308. doi: 10.1186/s12909-016-0809-8 (PMC5133756; doi:10.1186/s12909-016-0809-8)
Supplement: Additional file 1: — The Clinical Learning Environment and Supervision (CLES) in its adapted form, sent to medical students who had clinical practice in primary health care. [file 12909_2016_809_MOESM1_ESM.docx]

**Clinical Learning Environment and Supervision (CLES)** in its adapted form, sent to medical students who had clinical practice in primary healthcare

|  | How old are you? |  |
| --- | --- | --- |
|  | Do you have previous health education? |  |
|  | Which was you semester during spring 2012? |  |
|  | Which primary healthcare centre was your last clinical practice during spring 2012 |  |
|  | Are you: Woman. Man |  |
|  | Was your main supervisor: Woman. Man |  |
| CLES  items | The staff was easy to approach  1) Fully disagree. 2) Disagree to some extent. 3) Neither agree nor disagree. 4) Agree to some extent. 5) Fully agree. |  |
| CLES  items | I felt comfortable going to the PHC centre every day of my practice  1) Fully disagree. 2) Disagree to some extent. 3) Neither agree nor disagree. 4) Agree to some extent. 5) Fully agree. |  |
| CLES  items | During staff meetings I felt comfortable taking part in the discussions  1) Fully disagree. 2) Disagree to some extent. 3) Neither agree nor disagree. 4) Agree to some extent. 5) Fully agree. |  |
| CLES  items | There was a positive atmosphere at the PHC centre  1) Fully disagree. 2) Disagree to some extent. 3) Neither agree nor disagree. 4) Agree to some extent. 5) Fully agree. |  |
| CLES  items | The staff was generally interested in student supervision  1) Fully disagree. 2) Disagree to some extent. 3) Neither agree nor disagree. 4) Agree to some extent. 5) Fully agree. |  |
| CLES  items | The staff learned to know the students by their personal names  1) Fully disagree. 2) Disagree to some extent. 3) Neither agree nor disagree. 4) Agree to some extent. 5) Fully agree. |  |
| CLES  items | There were sufficient meaningful learning situations at the PHC centre  1) Fully disagree. 2) Disagree to some extent. 3) Neither agree nor disagree. 4) Agree to some extent. 5) Fully agree. |  |
| CLES  items | The learning situations were multidimensional in terms of content  1) Fully disagree. 2) Disagree to some extent. 3) Neither agree nor disagree. 4) Agree to some extent. 5) Fully agree. |  |
| CLES  items | The PHC centre can be regarded as a good learning environment  1) Fully disagree. 2) Disagree to some extent. 3) Neither agree nor disagree. 4) Agree to some extent. 5) Fully agree. |  |
| CLES  items | The manager of the PHC centre regarded the staff at their PHC centre as a key resource  1) Fully disagree. 2) Disagree to some extent. 3) Neither agree nor disagree. 4) Agree to some extent. 5) Fully agree. |  |
| CLES  items | The manager of the PHC centre was a team member  1) Fully disagree. 2) Disagree to some extent. 3) Neither agree nor disagree. 4) Agree to some extent. 5) Fully agree. |  |
| CLES  items | Feedback from the manager of the PHC centre could easily be considered as a learning situation  1) Fully disagree. 2) Disagree to some extent. 3) Neither agree nor disagree. 4) Agree to some extent. 5) Fully agree. |  |
| CLES  items | The effort of individual employees was appreciated  1) Fully disagree. 2) Disagree to some extent. 3) Neither agree nor disagree. 4) Agree to some extent. 5) Fully agree. |  |
| CLES  items | The PHC centre had a clearly defined vision and mission statement for the patient care that was clearly described  1) Fully disagree. 2) Disagree to some extent. 3) Neither agree nor disagree. 4) Agree to some extent. 5) Fully agree. |  |
| CLES  items | Patients received individualised care  1) Fully disagree. 2) Disagree to some extent. 3) Neither agree nor disagree. 4) Agree to some extent. 5) Fully agree. |  |
| CLES  items | There were no problems in the information flow related to patient care (Discussions about individual patients and the transmission of information  about individual patient cases to other colleagues and team members were handled respectfully)  1) Fully disagree. 2) Disagree to some extent. 3) Neither agree nor disagree. 4) Agree to some extent. 5) Fully agree. |  |
| CLES  items | Documentation of patient care (e.g medical records and other medical procedures etc) was clear  1) Fully disagree. 2) Disagree to some extent. 3) Neither agree nor disagree. 4) Agree to some extent. 5) Fully agree. |  |
| CLES  items | My supervisor showed a positive attitude towards supervision  1) Fully disagree. 2) Disagree to some extent. 3) Neither agree nor disagree. 4) Agree to some extent. 5) Fully agree. |  |
| CLES  items | I felt that I received individual supervision  1) Fully disagree. 2) Disagree to some extent. 3) Neither agree nor disagree. 4) Agree to some extent. 5) Fully agree. |  |
| CLES  items | I continuously received feedback from my supervisor  1) Fully disagree. 2) Disagree to some extent. 3) Neither agree nor disagree. 4) Agree to some extent. 5) Fully agree. |  |
| CLES  items | Overall I am satisfied with the supervision I received at the PHC centre  1) Fully disagree. 2) Disagree to some extent. 3) Neither agree nor disagree. 4) Agree to some extent. 5) Fully agree. |  |
| CLES  items | The supervision was based on a relationship of equality and promoted my learning  1) Fully disagree. 2) Disagree to some extent. 3) Neither agree nor disagree. 4) Agree to some extent. 5) Fully agree. |  |
| CLES  items | There was a mutual interaction in the supervisory relationship  1) Fully disagree. 2) Disagree to some extent. 3) Neither agree nor disagree. 4) Agree to some extent. 5) Fully agree. |  |
| CLES  items | Mutual respect and approval prevailed in the supervisory relationship  1) Fully disagree. 2) Disagree to some extent. 3) Neither agree nor disagree. 4) Agree to some extent. 5) Fully agree. |  |
| CLES  items | The supervisory relationship was characterized by a sense of trust  1) Fully disagree. 2) Disagree to some extent. 3) Neither agree nor disagree. 4) Agree to some extent. 5) Fully agree. |  |
