# Supplementary material for: Adaptation and validation of the instrument Clinical Learning Environment and Supervision for medical students in primary health care
Source: BMC Med Educ. 2016 Dec 1;16:308. doi: 10.1186/s12909-016-0809-8 (PMC5133756; doi:10.1186/s12909-016-0809-8)
Supplement: Additional file 2: Table S3. — The means and standard deviations of the items in the CLES instrument. [file 12909_2016_809_MOESM2_ESM.docx]

**Table 3 Means and Standard Deviations of the items in the CLES instrument Number of observations 349**

| Item | Mean | Std. Deviation |
| --- | --- | --- |
| Item1 | 4,60 | ,853 |
| Item2 | 4,34 | 1,063 |
| Item3 | 4,06 | 1,178 |
| Item4 | 4,34 | 1,054 |
| Item5 | 4,40 | ,947 |
| Item6 | 4,34 | 1,025 |
| Item7 | 4,52 | ,874 |
| Item8 | 4,32 | ,960 |
| Item9 | 4,53 | ,785 |
| Item10 | 4,37 | ,972 |
| Item11 | 3,51 | 1,137 |
| Item12 | 4,46 | ,762 |
| Item13 | 4,34 | ,949 |
| Item14 | 3,97 | 1,107 |
| Item15 | 4,04 | 1,085 |
| Item16 | 4,01 | 1,019 |
| Item17 | 4,38 | ,969 |
| Item27 | 3,98 | ,977 |
| Item21 | 3,79 | 1,127 |
| Item29 | 3,45 | 1,189 |
| Item30 | 3,92 | ,900 |
| Item31 | 3,53 | ,903 |
| Item32 | 4,46 | ,788 |
| Item33 | 4,53 | ,778 |
| Item34 | 4,51 | ,766 |

| Item 1 | My supervisor showed a positive attitude towards supervision |
| --- | --- |
| Item 2 | I felt that I received individual supervision |
| Item 3 | I continuously received feedback from my supervisor |
| Item 4 | Overall I am satisfied with the supervision I received at the PHC centre |
| Item 5 | The supervision was based on a relationship of equality and promoted my learning |
| Item 6 | There was a mutual interaction in the supervisory relationship |
| Item 7 | Mutual respect and approval prevailed in the supervisory relationship |
| Item 8 | The supervisory relationship was characterized by a sense of trust |
| Item 9 | The staffs was easy to approach |
| Item 10 | I felt comfortable going to the PHC centre every day of my practice |
| Item 11 | During staff meetings I felt comfortable taking part in the discussions |
| Item 12 | There was a positive atmosphere at the PHC centre |
| Item 13 | The staff was generally interested in student supervision |
| Item 14 | The staff learned to know the students by their personal names |
| Item 15 | There were sufficient meaningful learning situations at the PHC centre |
| Item 16 | The learning situation were multidimensional in terms of content |
| Item 17 | The PHC centre can be regarded as a good learning environment |
| Item 27 | The manager of the PHC centre regarded the staff at their PHC centre as a key resource |
| Item 28 | The manager of the PHC centre was a team member |
| Item 29 | Feedback from the manager of the PHC centre could easily be considered as a learning situation |
| Item 30 | The effort of individual employees was appreciated |
| Item 31 | The PHC centre has a clearly defined vision and mission statement for the patient care that is clearly described |
| Item 32 | Patients received individualised care |
| Item 33 | There were no problems in the information flow related to patient care (Discussions about individual patients and the transmission of information about individual patent cases to other colleagues and team members were handled respectfully) |
| Item 34 | Documentation of patient care (e.g medical records and other medical procedures etc) was clear |
