# Supplementary material for: Adaptation and validation of the instrument Clinical Learning Environment and Supervision for medical students in primary health care
Source: BMC Med Educ. 2016 Dec 1;16:308. doi: 10.1186/s12909-016-0809-8 (PMC5133756; doi:10.1186/s12909-016-0809-8)
Supplement: Additional file 3: Table S4. — The frequency of the Likert scale answers of the items in the CLES instrument. [file 12909_2016_809_MOESM3_ESM.docx]

|  | **Table 4. Frequency of the Likert scale answers of the items in the CLES instrument** | | | | | | | | | |  |
| --- | --- | --- | --- | --- | --- | --- | --- | --- | --- | --- | --- |
|  | Fully disagree | | Disagree to some extent | | Neither agree nor disagree | | Agree to some extent | | Fully agree | | Median |
|  | Freq | Percent | Freq | Percent | Freq | Percent | Freq | Percent | Freq | Percent |  |
| Item 1 | 7 | 1.78 | 15 | 3.81 | 9 | 2.28 | 65 | 16.50 | 298 | 75.63 | 5 |
| Item 2 | 15 | 3.81 | 23 | 5.84 | 19 | 4.82 | 95 | 24.11 | 242 | 61.42 | 5 |
| Item 3 | 21 | 5.33 | 36 | 9.14 | 27 | 6.85 | 123 | 31.22 | 187 | 47.46 | 4 |
| Item 4 | 12 | 3.05 | 27 | 6.85 | 21 | 5.33 | 88 | 225.34 | 246 | 62.44 | 5 |
| Item 5 | 11 | 2.79 | 11 | 2.79 | 30 | 7.61 | 100 | 25.38 | 242 | 61.42 | 5 |
| Item 6 | 13 | 3.30 | 18 | 4.57 | 30 | 7.61 | 93 | 23.60 | 240 | 60.91 | 5 |
| Item 7 | 8 | 2.03 | 8 | 2.03 | 29 | 7.36 | 77 | 19.54 | 272 | 69.04 | 5 |
| Item 8 | 9 | 2.28 | 13 | 3.30 | 45 | 11.42 | 101 | 25.63 | 226 | 57.36 | 5 |
| Item 9 | 3 | 0.76 | 12 | 3.05 | 18 | 4.57 | 102 | 25.89 | 259 | 65.74 | 5 |
| Item 10 | 5 | 1.27 | 29 | 7.36 | 23 | 5.84 | 96 | 24.37 | 241 | 61.17 | 5 |
| Item 11 | 24 | 6.09 | 31 | 7.87 | 162 | 41.12 | 75 | 19.04 | 102 | 25.89 | 3 |
| Item 12 | - | - | 16 | 4.06 | 17 | 4.31 | 129 | 32.74 | 232 | 58.88 | 5 |
| Item 13 | 8 | 2.03 | 21 | 5.33 | 22 | 5.58 | 123 | 31.22 | 220 | 55.84 | 5 |
| Item 14 | 15 | 3.81 | 38 | 9.64 | 42 | 10.66 | 146 | 37.06 | 153 | 38.83 | 4 |
| Item 15 | 10 | 2.54 | 46 | 11.68 | 25 | 6.35 | 149 | 37.82 | 164 | 41.62 | 4 |
| Item 16 | 10 | 2.54 | 30 | 7.61 | 52 | 13.20 | 156 | 39.59 | 146 | 37.06 | 4 |
| Item 17 | 9 | 2.28 | 22 | 5.58 | 18 | 4.57 | 107 | 27.16 | 238 | 60.41 | 5 |
| Item 27 | 5 | 1.27 | 4 | 1.02 | 150 | 38.07 | 71 | 18.02 | 164 | 41.62 | 4 |
| Item 28 | 16 | 4.06 | 19 | 4.82 | 146 | 37.06 | 63 | 15.99 | 150 | 38.07 | 4 |
| Item 29 | 35 | 8.88 | 21 | 5.33 | 171 | 43.40 | 64 | 16.24 | 103 | 26.14 | 3 |
| Item 30 | 3 | 0.76 | 7 | 1.78 | 138 | 35.03 | 118 | 29.95 | 128 | 32.49 | 4 |
| Item 31 | 4 | 1.02 | 30 | 7.61 | 184 | 46.70 | 107 | 27.16 | 69 | 17.51 | 3 |
| Item 32 | 1 | 0.25 | 18 | 4.57 | 13 | 3.30 | 127 | 32.23 | 235 | 59.64 | 5 |
| Item 33 | 1 | 0.25 | 13 | 3.30 | 25 | 6.35 | 91 | 23.10 | 264 | 67.01 | 5 |
| Item 34 | 1 | 0.25 | 6 | 1.52 | 42 | 10.66 | 87 | 22.08 | 258 | 65.48 | 5 |
|  |  |  |  |  |  |  |  |  |  |  |  |
|  |  |  |  |  |  |  |  |  |  |  |  |
