# Supplementary material for: Adaptation and validation of the instrument Clinical Learning Environment and Supervision for medical students in primary health care
Source: BMC Med Educ. 2016 Dec 1;16:308. doi: 10.1186/s12909-016-0809-8 (PMC5133756; doi:10.1186/s12909-016-0809-8)
Supplement: Additional file 4: Table S5. — The inter-factor correlations of the CLES instrument. [file 12909_2016_809_MOESM4_ESM.docx]

| **Table 5. Inter-Factor Correlations of the CLES instrument** | | | | | | | | |
| --- | --- | --- | --- | --- | --- | --- | --- | --- |
|  | **Factor1** |  | **Factor2** |  | **Factor3** |  | **Factor4** |  |
| **Factor1** | 100 | * | 56 | * | 62 | * | 31 | * |
| **Factor2** | 56 | * | 100 | * | 47 | * | 5 |  |
| **Factor3** | 62 | * | 47 | * | 100 | * | 21 |  |
| **Factor4** | 31 | * | 5 |  | 21 |  | 100 | * |
| **Printed values are multiplied by 100 and rounded to the nearest integer. Values greater than 0.3 are flagged by an '*'.** | | | | | | | | |

| Factor 1/Dimension 1 | Supervisor relationship |
| --- | --- |
| Factor 2/Dimension 2 | Pedagogical atmosphere on the PHC centre |
| Factor 3/Dimension 3 | Leadership style of the manager of the PHC centre |
| Factor 4/Dimension 4 | Premises of the patient |
